# Development of a Nutrient Profile Model for Dishes in Japan Version 1.0: A New Step towards Addressing Public Health Nutrition Challenges

**DOI:** 10.3390/nu16173012

**Published:** 2024-09-06

**Authors:** Yuko Tousen, Jun Takebayashi, Chika Okada, Mariko Suzuki, Ai Yasudomi, Katsushi Yoshita, Yoshiko Ishimi, Hidemi Takimoto

**Affiliations:** 1 National Institute of Health and Nutrition, National Institutes of Biomedical Innovation, Health and Nutrition, 3-17 Senrioka Shinmachi, Settsu-shi, Osaka 566-0002, Japan; tousen@nibiohn.go.jp (Y.T.); jtake@nibiohn.go.jp (J.T.); c-okada@nibiohn.go.jp (C.O.); suzuki-mariko@nibiohn.go.jp (M.S.); yasudomi@nibiohn.go.jp (A.Y.); 2 Department of Nutrition, Osaka Metropolitan University Graduate School of Human Life and Ecology, 3-3-138 Sugimoto, Sumiyoshi-ku, Osaka 558-8585, Japan; yoshita@omu.ac.jp; 3 Tokyo NODAI Research Institute, Tokyo University of Agriculture, 1-1-1 Sakuragaoka, Setagaya-ku, Tokyo 156-8502, Japan; yi207200@nodai.ac.jp

**Keywords:** nutrient profiling model, dishes, nutrients, Japanese food culture, public health nutrition, reformulation, dietary reference intakes

## Abstract

To address the rising incidence of non-communicable diseases (NCDs) and promote healthier eating habits, Japan requires a culturally tailored Nutrient Profile Model. This study aimed to develop a Nutrient Profile Model for Dishes in Japan version 1.0 (NPM-DJ (1.0)) that corresponds to the nutritional issues and food culture in Japan. The aim of the NPM-DJ (1.0) was to promote the health of the general population, and to prevent the increase in NCDs in Japan. The NPM-DJ (1.0) categorizes dishes into staples, sides, mains, mixed dishes, and mixed dishes with staples. The model evaluates dishes based on energy, saturated fats, sugars, and sodium as restricted nutrients, while considering protein, dietary fiber, and the weight of certain food groups as recommended nutrients. The distribution of the overall score for each dish category was analyzed and a rating algorithm was created. The baseline, modification points, and final scores were significantly lower for side dishes than for staple dishes. In contrast, the baseline points and final scores were significantly higher for mixed dishes with staple. The model effectively differentiated nutritional profiles across five dishes categories, which may promote healthier dish reformulation by food businesses operators and encourage consumers to select healthier dishes.

## 1. Introduction

Non-communicable diseases (NCDs) are responsible for approximately three-quarters of premature deaths globally, most of which are preventable through changes to modifiable risk factors [1]. Excess salt consumption is attributable for 1.65 million deaths annually worldwide [2,3]. The World Health Organization (WHO) has identified a 30% decrease in population salt intake as a significant objective within its global action plan for the prevention and management of NCDs and recommends a maximum dietary salt intake of less than 5 g/day for adults [4,5]. It is estimated that even a small downward shift in blood pressure at the population level leads to a large decrease in BP-related cardiovascular diseases [6]. In Japan, excessive salt intake is an important nutritional concern since the incidence of hypertension is 56.1% among men and 41.7% among women; moreover, cardiovascular disease is the fourth leading cause of death, accounting for 6.8% of all fatalities [7].

In Japan, the target of health policy is to “extend healthy life expectancy”, and the Health Japan 21 (Third Term) for Japanese people set a target salt intake of less than 7.0 g/day [8]. However, the average salt intake in 2019 was 10.1 g (10.9 g for men and 9.3 g for women), exceeding the target amount, and there has been no significant change in salt intake over the past 10 years [9]. Furthermore, in Japan, the main source of sodium intake is seasonings, such as salt, soya sauce, and miso, which are added to dishes (61.7% of the total sodium intake in men and 62.9% in women) [10]. Therefore, the public needs to reduce the amount of salt they consume from seasonings. In addition, the incidence of NCDs is increasing in Japan due to changes in lifestyles such as decreases in physical activity (decreased step count) and the shift to Western cuisine that is high in fat as well as increased meat intake [9]. The percentage of individuals with a blood cholesterol level of 240 mg/dL or higher is rising annually for both genders [7]. Japan lacks sufficient databases for saturated fatty acids and sugars, resulting in a lack of research in these areas to date. To reverse this trend, we need systems and food environments that allow people to choose healthier dishes in Japan. Consequently, it is necessary for the food business operators to implement mechanisms and systems that encourage the reformulation of recipes and products such as seasonings.

As part of a comprehensive policy response to promote healthy eating habits and to prevent disease, “nutrient profiles (NPs)”, which can comprehensively assess the nutritional value of foods, are used. The WHO defines nutritional profiling as “the science of classifying or ranking foods according to their nutritional composition for reasons related to preventing disease and promoting health” [11]. Countries such as the UK and France have successfully implemented nutrient profile models to guide public health policies [12,13,14,15]. In Europe, consuming foods with low NP model ratings is linked to higher mortality from all causes, including cancer and cardiovascular, respiratory, and digestive diseases [16]. In Chile, mandatory front-of-package warning labels on unhealthy foods and beverages have significantly reduced sugar and sodium contents in packaged products [17]. NP has also been used in Asian countries, such as Singapore and India, the USA, and Canada to limit front-of-pack food labeling, restrict health claims, and regulate advertising aimed at children [18,19,20,21].

However, these existing NPs were formulated mainly for Western processed foods and/or meals. In Asian countries, including Japan, meals consist of “dishes” that combine multiple foods flavored using traditional seasonings [22,23], and compared to meals from Western countries, Japanese meals, which combine these dishes, are low in fat, animal protein, and sugar but high in sodium [9,24]. As mentioned above, especially regarding the dietary sources of salt among Japanese people, a large proportion is consumed through cooking seasonings at home [25,26], a trait characteristic of the Japanese diet. A high percentage of Japanese people ate meals at home: 80.7% ate breakfast, 56.8% ate lunch, and 90.7% ate dinner [7]. Therefore, it is essential to present a “dish version of NPs”, for the Japanese diet, which has not been attempted in other countries. In other words, it is necessary to develop an NP for dishes that are feasible in Japan and consider the Japanese food culture.

## 2. Materials and Methods

### 2.1. Scope of the Nutrient Profile Model for Dishes in Japan Version 1.0 (NPM-DJ (1.0))

The NPM-DJ (1.0) was developed from the perspective of public nutrition in Japan, targeting people aged 18 years or older, with the primary goal of promoting health in the general population and preventing the increase in NCDs in Japan based on the evaluation of dishes typically consumed by Japanese people. The NPM-DJ (1.0) is also intended for use by food business operators as a guideline for improving the quality of products/dishes and changing their formulations, as well as by consumers as a guideline for cooking at home and selecting healthier dishes.

### 2.2. Deciding the Target Dishes, and Their Recipes, and Calculating Nutrient Composition

The target dishes for NPM-DJ (1.0) were classified as staple dishes, side dishes, or main dishes in the serving (SV) Quick Reference Table of the Japanese Food Guide Spinning Top (Ministry of Health, Labor, and Welfare and Ministry of Agriculture, Forestry, and Fisheries) [27,28]. First, the main foods in the dishes and their weights listed in the SV Quick Reference were used as recipes. Regarding the types and weights of other foods and seasonings required to make a dish that is not listed in the SV Quick Reference, three nutritionists and registered dietitians created and checked standard Japanese recipes from books on cooking dishes [29,30]. The books used to create the recipes contained basic information about dishes and home-cooked dishes that are commonly consumed in Japan, such as recipes, foods used in the recipes, weight of the food and seasoning, waste rate of each food item, and nutritional value [29,30]. Furthermore, the nutritionists and registered dietitians who verified the recipes all had experience creating menus for hospitals and other catering facilities, and they verified the recipes for this study based on standards that conformed to the nutrition and food composition based on the Dietary Reference Intakes for Japanese [31].

The nutritional value of the recipes was calculated using nutritional value calculation software (Excel add-in “Nutrition Plus®”, Kenpakusha, Tokyo, Japan) based on the 2020 edition (8th revision) of the Standard Tables of Food Composition in Japan [32]. Nutrients in the dishes were calculated by assuming the state and amount at the time of consumption after cooking. For the foods listed in the Standard Tables of Food Composition in Japan, the values after cooking were used, and the salt and oil absorption rates were also reflected [29,30,32]. The salt and oil absorption rates were calculated based on information from a book that listed the actual values of salt and oil absorption rates based on recipe methods, cooking times, ingredients, etc. [29,30].

### 2.3. Selecting the Classification of Dishes and Their Units

The dishes were classified according to the classification criteria of the Japanese Food Guide Spinning Top, and data from the National Health and Nutrition Survey was also used to consider classification based on the actual dietary situation in recent years, which follows a system of staples, sides, and main dishes [7,27,28]. As shown in Table 1, dish categorization was conducted in the order of steps 1 and 2. In the first step, dishes were classified as staple dishes if they contained more than two-thirds rice, bread, noodles, or other grains of total weight of the dish; side dishes if they contained more than two-thirds vegetables, potatoes, legumes other than soybeans, mushrooms, seaweed, nuts, and seeds; and main dishes if they contained more than two-thirds meat, fish, eggs, soybeans, or soybean products. One serving (SV) of the staple dish contained 40 g of carbohydrates derived from the main ingredient (rice, bread, noodles, or other grains), one SV of the side dish contained 70 g of the main ingredient (vegetables, mushrooms, potatoes, seaweed), and one SV of the main dish contained 6 g of protein derived from the main ingredient (meat, fish, eggs, soy products). The cutoff values of the first step were the same as the Japanese Food Guide Spinning Top [27]. Mixed dishes with a staple are combinations of staple with side or main dishes, as shown in Table 1. Although the Japanese Food Guide Spinning Top showed these as popular dishes in Japan, the main ingredient of the dish did not exceed two-thirds of total dish weight, not matching the criteria for dish categorization [27]. Therefore, we decided to create another category called “mixed dishes”, and further categorize them by the existence of staples. Staples such as rice, bread, or noodles are valued as an important component in a Japanese meal, and the government promotes meals consisting of staple, side, and main dishes as a balanced diet [8]. Thus, in the second step, a dish with a staple food with an SV of less than 0.5 and both a main dish and a side dish with an SV of 0.5 or more were classified as mixed dishes. A dish with a staple food with an SV of 0.5 or more and at least one main or side dish with an SV of 0.5 or more was classified as a mixed dish with a staple. The cutoff values of second step were from the results of the National Health and Nutrition Survey; it was possible to separate mixed dishes and mixed dishes with staple, focusing on staple dish content [7].

### 2.4. Target Dishes and Their Classification

The dishes listed in the Japanese Food Guide Spinning Top SV table were classified according to the rules of dish classification in NPM-DJ (1.0) (Table 1) [27]. There were 20 staple dishes, 34 side dishes, 21 main dishes, 12 mixed dishes (1), and 18 mixed dishes with staples (2), totaling 105 dishes.

### 2.5. Model Selection

**Figure 1:**
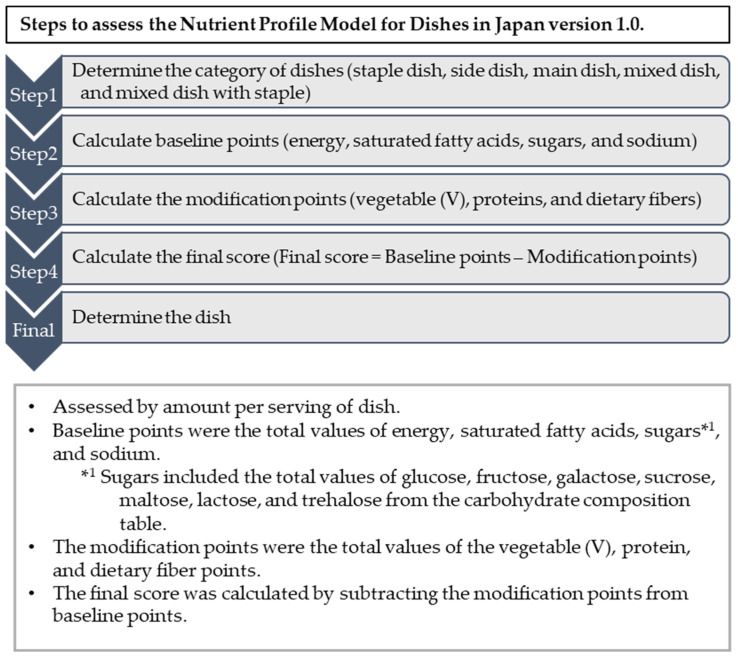
Steps to assess the Nutrient Profile Model for Dishes in Japan version 1.0.

We selected a scoring model based on Processed Foods in Japan version 1.0 (NPM-PFJ (1.0)) [33]. The steps for evaluating the NPM-DJ (1.0) are shown in Figure 1.

### 2.6. Model Algorithm

Our algorithm was based on NPM-PFJ (1.0) [33].

#### 2.6.1. Dish Units

In this study, food was assessed at the time of consumption and, ultimately, the evaluation was performed per SV.

#### 2.6.2. Selection of Nutrients and Food Groups

Nutrients included in the NPM-DJ (1.0) were selected from nutrients or food groups that are in excess or insufficient for public nutrition in Japan. In other words, energy, saturated fatty acids, sugars, and sodium were selected as the nutrients whose excessive intake could affect health. The total weight of cooked fruits, vegetables, nuts, legumes, seaweed, seeds, and mushrooms, as well as nutrients such as protein and dietary fiber, were selected as components whose insufficient intake could affect health (Figure 1 and Table S1).

#### 2.6.3. Baseline Points

The algorithm of the NPM-DJ (1.0) model is based on NPM-PFJ (1.0) [33], which is based on the HSR algorithm [34,35]. NPM-PFJ (1.0)’s algorithm has been modified based on the values of the Dietary Reference Intakes for Japanese and the Public Health Guideline, Health Japan 21 (Third Term) [8,31]. The restricted nutrients based on the baseline points and the basis for setting their algorithms are as follows:

[Energy]

Energy (kcal): 2200 kcal, score band started at 3.75% of the nutrient labeling standard value (Food Labeling Standards, Appendix 10) based on the Dietary Reference Intakes for Japanese (2020 edition) [31,36]. The figure of 3.75% follows the UK FSA NP Model 2004/5 [37], which is thought to be the basis of the Health Star Rating (HSR) algorithm [34,35].

[Saturated fatty acids]

Score band started at 7% of the energies in food based on the target intake of saturated fatty acids in the Dietary Reference Intakes for the Japanese (2020 edition) [31].

[Sugars]

Score band started at 10% of energy content (guideline: sugar intake for adults and children (4 March 2015, WHO)) [38].

[Sodium]

Score band started at 3.75% of the target value for salt intake in Health Japan 21 (Third Term) is 7 g (2756 mg as sodium) [8]. The upper limit from one point onwards was allocated equally in the same range as the upper limit at zero points.

#### 2.6.4. Modification Points

The modification points were the sum of the V, protein, and dietary fiber points. The score (final score) was calculated by subtracting the modification points from baseline values (Table S1). The algorithm of the NPM-DJ (1.0) model is based on NPM-PFJ (1.0) [33], which is based on the HSR algorithm [34,35]. NPM-PFJ (1.0)’s algorithm has been modified based on the values of the Dietary Reference Intakes for Japanese and the Public Health Guideline, Health Japan 21 (Third Term) [8,31]. The recommended nutrients based on the modification points and the basis for setting their algorithms are as follows:

[Vegetable (V)]

Vegetable (V) points scored between 0–8 according to the non-concentrated fvnl (%) per dish, as shown in Table S1. The fvnl was the total weight of cooked fruits, vegetables, nuts, and legumes, including seaweed, seeds, and mushrooms/total weight of cooked food (excluding water and stock). If the dish contained less than 40% fvnl, the V-point score was 0. Potatoes were not included in the V point calculation because they are rich in carbohydrates and also not counted as vegetables in the Japan National Health and Nutrition Survey [7].

[Protein]

Adjusted (2–15 points, weighted average with the HSR value). The score band started at 3.75% of 81 g (nutrient reference value (NRV)s (Japan, 2015)) and was adjusted (2–15 points, weighted average with the HSR value) [39].

[Dietary fiber]

Extended linearly (1–5 points) and adjusted (6–15 points, weighted average with the HSR value). The score band started at 3.75% of 19 g (NRVs (Japan, 2015)) and extended linearly (2–5 points) and was adjusted (6–15 points, weighted average with the HSR value) [39].

#### 2.6.5. Protein Cap Calculation Algorithm, Score Standard Value, and Score Table Setting

**Figure 2:**
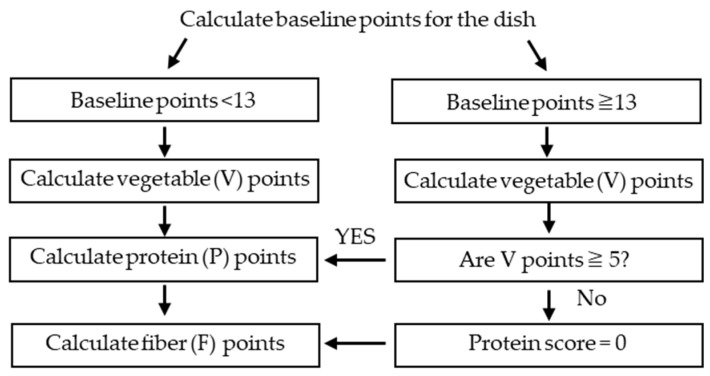
Protein cap calculation algorithm of the Nutrient Profile Model for Dishes in Japan version 1.0.

The calculation algorithm for the protein cap (products that score ≥ 13 baseline points are not permitted to score points for protein unless they score five or more V points) of nutrients is shown in Figure 2. The calculation algorithm of the score table and protein caps in NPM-DJ (1.0) were based on NPM-PFJ (1.0) [33]. The score table for nutrients and food groups was set based on the Dietary Reference Intakes for Japanese (2020 edition) [31] (Table S1).

#### 2.6.6. Final Score and Rating Algorithm

The scoring algorithm is illustrated in Figure 1. The baseline points were the sum of energy, saturated fatty acids, sugars, and sodium. For sugars, the sums of glucose, fructose, galactose, sucrose, maltose, lactose, and trehalose from the carbohydrate composition table in the 2020 edition (8th revision) of the Standard Tables of Food Composition in Japan were used [32]. Final scores = baseline points − modification points. The ratings were calculated by classifying the distribution of final scores for each dish category into 10 percentiles.

### 2.7. Statistical Analysis

The NPM-DJ scores (1.0) are presented as medians, interquartile ranges, and 95% confidence intervals of the population mean. Differences across dish categories were evaluated using the nonparametric Kruskal–Wallis test, followed by multiple comparisons using the Steel test. Staple dishes were used as the control group and their scores were tested to determine whether they were significantly higher or lower than those of the staple dishes. All analyses were performed using the statistical software package IBM SPSS Statistics version 25.0 (IBM Japan Ltd., Tokyo, Japan).

## 3. Results

### 3.1. Point Distribution by Dish Category: Baseline Points

**Figure 3:**
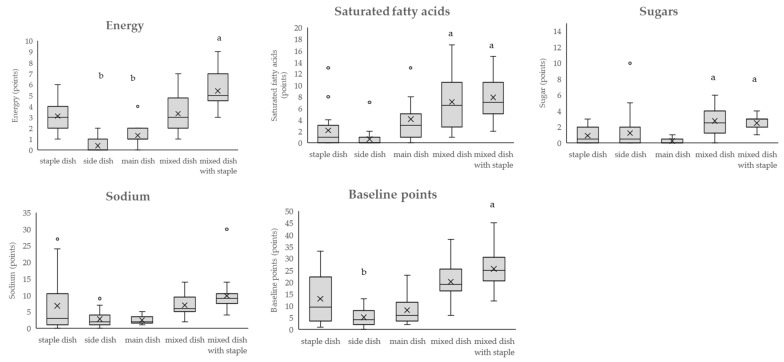
The distribution of energy, saturated fatty acid, sugar, sodium, and baseline points using the NPM-DJ (1.0). The number of each category is as follows: staple dish = 20; side dish = 34; main dish = 21; mixed dish = 12; and mixed dish with staple = 18. ○: outside the box indicates an outlier. ×: inside the box indicates the average (excluding the outlier). The top, middle, and bottom of the box indicate the third (75th percentile), second (median), and first (25th percentile) quartiles, respectively. The upper bar of the box plot indicates the maximum value and the lower bar indicates the minimum value. Outliers were values that were more than 1.5 times the first or third quartile range. Comparisons were made between all dish categories using the nonparametric Kruskal–Wallis test, followed by multiple comparisons using the Steel test. Staple dish was used as the control group, and tests were conducted to determine whether the values were significantly higher or lower than those of the staple dish. a, b: values with letters were significantly different from the staple dish. Statistical significance was set at a *p*-value of less than 0.05.

Figure 3 shows the point distributions of energy, saturated fatty acids, sugars, and sodium, and baseline points by dish category. For energy, the median point for the entire category was 1, with a range from 0 to 9 (95% confidence interval of the population mean: 1.85–2.69), and the minimum being side dishes and the maximum being a mixed dish with a staple (pork cutlet bowl). Compared with staple dishes, side and main dishes had significantly lower values, and mixed dishes with staples had significantly higher values. For the energy point, the outliers for the main dishes were pork cutlet (with sauce) (the score for the dish: 4) and beef steak (4). Pork cutlet (with sauce) has energy from the pork loin fat, and the pork is coated in batter and fried in oil, which increases its energy content. Beef steak had a lot of energy from beef. One of the reasons for the outliers was that the total food weight of the dish was high even within the main dish category (pork cutlet (with sauce): 123 g, beef steak: 157 g, median for main dish classification 62 g). That is, dishes that are large in overall volume, contain a high proportion of meat, and are deep-fried were outliers in the main dish category. Mixed dishes with staples contained a protein source from meat, fish, eggs, and vegetables in addition to the carbohydrate source (staple dishes: rice, noodles, and bread); hence, the whole weight of the dishes (excluding water and stock) was increased (median 329 g), increasing the energy content also. Side dishes were mainly made of low-energy foods, such as vegetables and seaweed, and the portions of side dishes are small in Japanese meals. Therefore, in this study, the weight of the dishes (excluding water and stock) (median 111 g) and the energy content were low.

For saturated fatty acids, the median score for the entire category was 2, with a range from 0 to 17 (95% confidence interval for the population mean: 2.75–4.11), and the minimum being side dishes (multiple dishes) and the maximum being a mixed dish (sukiyaki). Mixed dishes and dishes with staples had significantly higher scores than staple dishes. Among fatty acids, the outliers in the staple dish category were croissants (13) and chicken rice (8), while in the side dish category, it was butter-fried mushrooms (7), all of which had high levels of saturated fatty acids from butter. In the main dishes category, the pork cutlet (with sauce) (13) and beef steak (13) were outliers with high levels of saturated fatty acids in both the pork and beef. Scrambled eggs (omelet) (13) were also an outlier, with high levels of saturated fatty acids from the eggs and butter. In Japanese dishes in this study, dishes that use a lot of butter had high saturated fatty acid points in every category.

For sugars, the median score for the entire category was 1, with a range from 0 to 10 (95% confidence interval for the population mean: 1.02–1.66), and the minimum being side dishes (multiple dishes) and the maximum being a side dish (boiled pinto beans). Mixed dishes and dishes with staples had significantly higher scores than staple dishes. Simmered sweet pinto bean (10), which has sugar from pinto beans and refined sugar, was an outlier in the side dish category.

Regarding the sodium points, the outlier in the staple dish category was roasted pork ramen (with all of the soup consumed) (27), the outlier in the side dish category was root vegetable soup (miso soup with a lot of toppings) (9), and the mixed dish with a staple was Tenshin noodles (crab omelet on ramen, with all of the ramen soup consumed) (30), all of which were dishes that contained soup. As shown in Table S2, ramen soup has a high sodium content per dish. Roasted pork ramen had very high sodium points because it has roasted pork, which is seasoned with soy sauce and other seasonings on top of the noodles, and Tenshin noodles have toppings and a soup with seasoning on top of the ramen. Mixed dishes with staples contained a carbohydrate source (rice, noodles, and bread) as well as a protein source and vegetables (dish number 88–101 in Table S2. The condiments used to flavor these dishes such as soy sauce and the noodle broth both contributed to the sodium points. Side dishes are typically served in small portions in Japanese meals, so in this study the weight of the dishes (excluding water and stock) was low (median 111 g) and the sodium content was also relatively low.

Regarding baseline points, the median point value for the entire category was 9, with a range from 0 to 45 (95% confidence interval of the population mean: 10.38–14.31), and the minimum point being side dishes (lettuce and cucumber salads) and the maximum point being mixed dishes with staples (Tenshin noodles [crab omelet on ramen, with all of the ramen broth consumed]). Compared with staple dish, side dishes had significantly lower scores, whereas mixed dishes with staples had significantly higher scores.

### 3.2. Point Distribution by Dish Category: Modification Points

**Figure 4:**
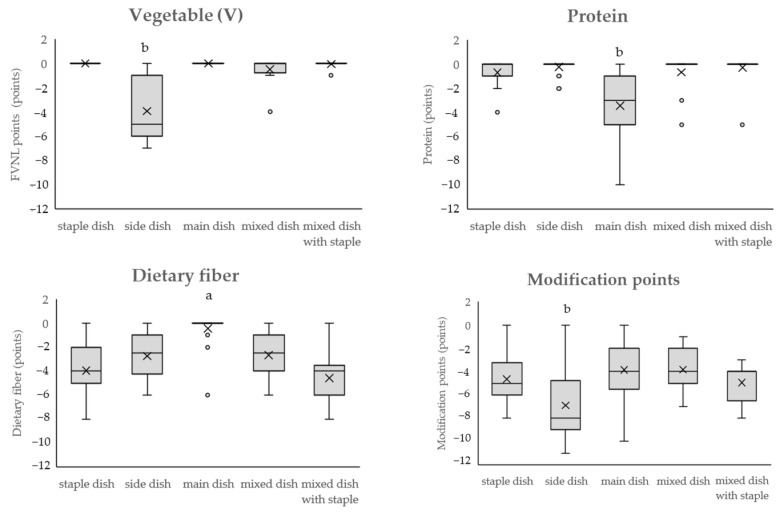
The distribution of vegetable (V), protein, dietary fiber, and modification points using the NPM-DJ (1.0). The number of dishes in each category is as follows: staple dish = 20; side dish = 34; main dish = 21; mixed dish = 12; and mixed dish with staple = 18. Modification points are positive points; however, because they are subtracted from the baseline points to calculate the final score, they are shown as negative points. ○: outside the box indicates an outlier. ×: inside the box indicates the average (excluding the outlier). The top, middle, and bottom of the box indicate the third (75th percentile), second (median), and first (25th percentile) quartiles, respectively. The upper bar of the box plot indicates the maximum value and the lower bar indicates the minimum value. Outliers were values that were more than 1.5 times the first or third quartile range. Comparisons were made between all dish categories using the nonparametric Kruskal–Wallis test, followed by multiple comparisons using the Steel test. Staple dishes were used as the control group, and tests were conducted to determine whether the values were significantly higher or lower than those of the staple dish. a, b: values with letters were significantly different from the staple dish. Statistical significance was set at a *p*-value of less than 0.05.

Figure 4 shows the point distribution by dish category for the V, protein, and dietary fiber, and modification points. The modification points are essentially positive points; however, because they are subtracted from the baseline points to calculate the final score, they are considered negative points. The median V points across all categories were 0, with a range from −7 to 0 (95% confidence interval of the population mean: −1.80–−0.90). The minimum was for side dishes and the maximum was observed in multiple categories. Side dishes scored significantly lower than staple dishes. In the mixed dish category, cabbage rolls (4) were outliers, and in the mixed dish with staple category, yakisoba (fried noodles) (1) and fried gyoza (with sauce) (1) were outliers. In these categories, most dishes had a V point of 0; therefore, even a V point of 1 was an outlier. 

For proteins, the median score for the entire category was 0, with a range from −10 to 0 (95% confidence interval for the population mean: −1.39 to −0.65). The minimum score was for side dishes (multiple dishes) and the maximum score was for main dishes (karaage [Japanese-style fried chicken]). The number of main dishes was significantly lower than that of staple dishes. On the protein point, tendon (tempura on rice with sauce) (4) in the staple dish category, seaweed and tuna salad (2), salted boiled edamame (green soy beans (1), boiled broccoli salad (1), Chinese-style stir-fried spinach (1), simmered kiriboshi daikon (1), corn soup (1), and root vegetable soup (miso soup with lot of toppings) (1) in the side dish category, and simmered deep-fried vegetable tofu (3) and deep-fried horse mackerel marinated in sweet vinegar sauce (5), and takoyaki (octopus balls) in the mixed dish with staple category were outliers. Because dishes with protein as the main ingredient are classified as main dishes, fish and shellfish, soy products (fried tofu and miso), and products containing milk, which contain protein, were outliers in every dish category.

Regarding dietary fiber, the median point for the entire category was −3, with a range from −8 to 0 (95% confidence interval for the population mean: −3.26 to −2.36), with the minimum being side dishes (multiple dishes) and the maximum being staple dishes and mixed dishes with staples (multiple dishes). The number of main dishes was significantly higher than that of staple dishes. On the dietary fiber point, in the main dish category, natto (6), Mapo tofu (2), and chilled tofu (1) were outliers, and all other dishes had 0 points. These three dishes were made primarily with soy products.

Regarding the modification points, the median point value for the entire category was −5, with a range from −11 to 0 (95% confidence interval for the population mean: −4.64 to −5.72). The minimum was for side dishes (multiple dishes) and the maximum was for staples, sides, and main dishes (multiple dishes). The number of side dishes was significantly higher than that of staple dishes. No outliers were found.

**Figure 5:**
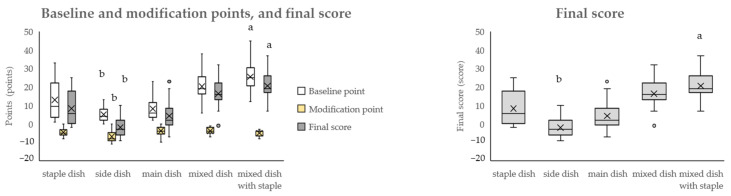
The distribution of baseline, modification, and final points using the NPM-DJ (1.0). The number of dishes in each category is as follows: staple dish = 20; side dish = 34; main dish = 21; mixed dish = 12; and mixed dish with staple = 18. Modification points are positive points; however, because they are subtracted from the baseline points to calculate the final score, they are shown as negative points. ○: outside the box indicates an outlier. ×: inside the box indicates the average (excluding the outlier). The top, middle, and bottom of the box indicate the third (75th percentile), second (median), and first (25th percentile) quartiles, respectively. The upper bar of the box plot indicates the maximum value and the lower bar indicates the minimum value. Outliers were values that were more than 1.5 times the first or third quartile range. Comparisons were made between all dish categories using the nonparametric Kruskal–Wallis test, followed by multiple comparisons using the Steel test. Staple dishes were used as the control group, and tests were conducted to determine whether the values were significantly higher or lower than those of the staple dishes. a, b: values with letters were significantly different from the staple dishes. Statistical significance was set at a *p*-value of less than 0.05.

Figure 5 shows the baseline and modification points and final scores for each dish category. The modified points were subtracted from the baseline points to calculate the final score. The baseline, modification points, and final scores were significantly lower for side dishes than for staple dishes. In contrast, the baseline points and final scores were significantly higher for mixed dishes with staple than for those with staple dishes. The median point for the final score across all categories was 3, with a range from −9 to 37 (95% confidence interval of the population mean: 5.05–9.28), with the minimum score being for side dishes (salted boiled edamame [green soybeans], boiled broccoli salad, and shungiku [garland chrysanthemum] with sesame sauce) and the maximum score being for mixed dishes with staples (Tenshin noodles [crab omelet on ramen, with all of the ramen broth consumed]). Compared with staple dishes, the scores of side dishes were significantly lower and mixed dishes with staple were significantly higher. For the final score, the outliers for the main dishes were pork cutlet (with sauce) (23). Pork cutlet (with sauce) is high in energy, saturated fat and sodium points. Simmered deep-fried vegetable tofu in the mixed dish with staples category was an outlier because baseline points were low and it had V, protein, and dietary fiber points. The baseline points of mixed dishes with staples were high and modification points were low, resulting in high final scores. High baseline points (≥13) lead to zero points for protein scores (Figure 2), which is the reason for the high final scores. For example, the fried pork cutlet bowl with egg dish has a deep-fried piece of pork cooked together with eggs and flavored with soy sauce and sugar on top of boiled rice. As a result, energy, saturated fatty acids, sugars, and sodium values are high.

### 3.3. Rating within the Dish Category

The final scores were classified into the 10th percentile for each dish category: staple dish, side dish, main dish, mixed dish, and mixed dish with staple (Table 2). There were only 12 types of mixed dishes and 18 types of mixed dishes with staples; therefore, there was bias in the distribution. For mixed dishes with staples, the range of ratings from 2.5 to 4.0 was included between a score of 17 and 19, indicating that the rating results differed even for the same final score.

### 3.4. The Effect of Noodle Soup Consumption on Final Scores

Table 3 shows the effect of consuming different amounts of noodle soup (left all soup, consumed half soup, and consumed all soup) on the final score. For udon (thick wheat noodles) and tempura udon, if all of the broth was left behind, the final score was 9 points lower than if the whole was consumed. For zaru soba (cold buckwheat noodles), if all of the broth was left behind, the final score was 12 points lower than if the whole was consumed. For ramen (soy sauce), if all of the broth was left behind, the final score was 22 points lower than if the whole was consumed. The reason for the large change in points for zaru soba and ramen is that in the protein cap calculation algorithm, the protein point score changes depending on whether the baseline point is less than 13. For roasted pork ramen and Tenshin noodles (crab omelet on ramen), if all of the broth was left behind, the final scores were 17 and 12 points lower, respectively, than if consumed completely.

### 3.5. Scores and Ratings of Dishes Listed in the Dietary Guide Consolidated List

Table 4 shows the scores and ratings of dishes listed in the Dietary Guide SV Quick Reference Table evaluated using the NPM-DJ (1.0). In addition, recipe information is provided for the 105 dishes included in this study (Supplemental Table S2).

## 4. Discussion

In this study, we developed the NPM-DJ (1.0) by expanding our previously created NPM-PFJ (1.0) into a model that can be applied to a dish. This model assesses dishes based on Japanese eating habits and food culture and is a system that can suggest appropriate ways to eat by combining foods and seasonings. This NP model was created independently of any food business operator, and its utilization by food business operators may lead to the reformulation of the nutritional content of dishes (product cuts) and encourage consumers to select healthier dishes and may be a solution to Japan’s future nutritional challenges.

The WHO has expressed concern that NP standards should be developed in relation to the public nutritional problems, culture, and circumstances of each country and has expressed concern about whether NP standards can be applied to other cultures and purposes [11]. Japanese food culture is unique, and traditional meals consist of “dishes” that combine multiple foods, and a meal consists of a combination of dishes [22,40,41]. The NPM-DJ (1.0) classifies dishes into staple dishes, side dishes, main dishes, mixed dishes, and mixed dishes with staple, considering Japan’s food culture and eating habits. Staple dishes are mainly made up of grains and consist of rice, bread, and noodles. Side dishes are dishes centered on vegetables, seaweed, and mushrooms; main dishes are dishes centered on meat, fish, and soy products; and there are mixed dishes that combine staple dishes, side dishes, and main dishes. Carbohydrates (main source is rice, bread, and noodles) account for approximately 60% of the energy ratio in Japanese meals, and meals can be eaten as a combination of a staple dish, side dish, and main dish, or as a combination of all three in one dish (Japanese-style curry and rice, fried pork cutlet bowl with egg, yakisoba (fried noodles)) [7]. Although the Japanese Food Guide Spinning Top showed these dishes as popular ones in Japan, the main ingredient of the dish did not exceed two-thirds of total dish weight, not matching the criteria for dish categorization [27]. Therefore, we decided to create another category called “mixed dishes”, and further categorize them by the existence of staples. Staples such as rice, bread, or noodles are valued as an important component in a Japanese meal, and the government promotes meals consisting of staple, side, and main dishes as a balanced diet [8]. Japanese meals, which combine these dishes, are low in fat, animal protein, and sugar intake, but high in sodium intake, compared with those in Western countries [7,24]. In the assessment using the dish category of the NPM-DJ (1.0), the distribution of the baseline points, modification points, and the final scores (Figure 3, Figure 4 and Figure 5) showed the characteristics of the dishes in each category. For example, croissants and chicken rice (staple dishes), which contain butter in their recipes, and beef steak (main dish), which uses beef fat, have notably high saturated fatty acid points in each dish category. Dishes using soy, such as natto, chilled tofu, and Mapo tofu, had dietary fiber points in the main dishes.

The NPM-DJ (1.0) evaluates each dish using a standardized unit (SV) at the point when the dish is ready to be eaten, and the considered. The nutrient profiles of processed foods reported thus far are often evaluated per 100 g or 100 kcal, which is affected by the moisture content of the food, making it difficult to evaluate the state and amount of food at the time of consumption (e.g., dried or salted) [34,42,43]. Since traditional seasonings such as soy sauce and miso are consumed as an ingredient in a dish [26,44], we decided to evaluate the total of a dish in the NPM-DJ (1.0) by including them together. In Japan, one of the recommended ways to reduce salt intake is to “leave the soup in noodles [45]”. However, although health guidance materials provided by registered dietitians include information about ramen soup, this information is not known to the average consumer. Our study is the first to demonstrate its salt reduction effect using nutritional profiles. It is easy for consumers to understand that whether one consumes broth from udon, soba, or ramen noodles affects its final NP score, demonstrating that this NP is useful for suggesting eating habits and may even be useful in reducing salt intake in nutritional policies. In Japan, it is popular to eat noodles in broth, topped with various cooked foods. Although the samples presented in this study are limited, they cover the majority of these noodle dishes in Japan [46].

The dishes presented in this study, which are limited to the 105 dishes listed in the Japanese Food Guide Spinning Top, do not completely represent the diversity of cooking methods across Japan, and only standard Japanese dishes have been proposed [27]. In addition, the nutritional data and seasonings are based on the Dietary Reference Intakes for Japanese, and the dishes are likely to contain less salt, oil, and sugar than typical ready-meals or restaurant dishes [31]. To further improve the accuracy and usefulness of the NPM-DJ (1.0), more data on cooking recipes that reflect the actual food culture and eating habits in Japan are needed.

The application of the NPM-DJ (1.0) system can lead to a change in product formulation (1); improve the nutritional quality of a product, such as ready-made meals, home cooking and restaurant dishes (2); and impact the Japanese population’s dietary intake (3), thereby improving the nutritional status of the people and public health. An NP-driven 5-year voluntary reformulation strategy was reported to have led to a decrease in sodium and added sugars across eight food and beverage categories in the USA and France [47]. Going one step further, an analysis using reformulation scenarios and a health model showed that the total impact of reformulation resulted in avoided deaths per year in France, equivalent to a 3.7–5.5% reduction in mortality linked to diet-related chronic diseases including coronary heart disease, stroke, and some cancers [48].

The goal of nutritional profiling is to evaluate foods and dishes based on their nutritional content and food composition. Currently, many NP systems have been developed around the world, including government models (Nutri-Score (France and other European regions)), HSR (Australia, New Zealand), keyhole (Nordic countries), Health Promotion Board (Singapore), etc. and models developed by food business operators [14,16,18,34,44,47,49]. The NPM-DJ (1.0) has not been validated with other NP models. However, except for the food classification, the calculation table, protein cap calculation algorithm, and score table of the NPM-DJ (1.0) are the same as those of the NPM-PFJ (1.0) [33]. The NPM-PFJ (1.0) was created with reference to the HSR and shows a high correlation with the HSR, and its validity has been verified [33]. Recently, in a systematic review examining the criteria for nine NP systems, Barrett et al. reported that Nutri-Score NPS was assessed as having substantial criterion validation evidence, and the Food Standards Agency NPS, Health Star Rating, Nutrient Profiling Scoring Criterion, Food Compass, Overall Nutrition Quality Index, and the Nutrient-Rich Food Index were determined as having intermediate criterion validation evidence [50]. In addition, a study in Australia suggested that products displaying an HSR have a lower sodium content and that food manufacturers are more likely to display an HSR on healthy products if they voluntarily use the HSR [51]. Therefore, the NPM-DJ (1.0) may lead to voluntary improvement in salt-reduced products (dishes) in the food business operators.

The NP scores in this study were for the standard amount of each dish per serving. The food weight of each dish is shown in Supplementary Table S2 and the weight of the entire dish is shown in Table 4. As the overall amount of a dish increases, the amount of nutrients and food naturally increases; therefore, each dish does not necessarily meet the NP evaluation results shown in this study. Moreover, the NPM-DJ (1.0) score was created based on the basic recipes of Japanese dishes and could not provide any indication of the healthiness of the dish indicated by the score range in the present study. Although the NPM-DJ (1.0) model aligns with Dietary Reference Intakes for Japanese and dietary guidelines, there is a need for further research on the true effect of implementing the model on population health. In the future, the relationship between the NPM-DJ (1.0) score, which is based on food and nutrient intake, and health indicators (body mass index, blood pressure, blood lipid levels, blood glucose levels, etc.) based on the actual results of the Japan National Health and Nutrition Survey should be examined to demonstrate the healthiness of the score.

Although reformulated food products may have improved nutrient profiles, ultimately, the final test of a successful product reformulation is whether or not the reformulated foods are actually purchased and consumed [52]. According to the National Health and Nutrition Survey conducted in Japan, approximately 60% of men and women who consume 8 g or more of salt per day do not intend to improve their dietary habits [7]. National salt reduction strategies were identified in 75 countries in 2015; however, the debate regarding the most effective and acceptable salt reduction strategy continues [53,54]. Several systematic reviews on strategies to improve diets have suggested that food product reformulation would effectively reduce sodium intake [54,55]. Hyseni L et al. reported that comprehensive strategies involving multiple components (reformulation, food labeling, and media campaigns) and “upstream (regulatory and fiscal interventions)” population-wide policies such as mandatory reformulation generally appear to achieve larger reductions in population-wide salt consumption than “downstream (dietary counselling (for individuals, worksites or communities))”, individually focused interventions [54]. Therefore, reducing salt intake throughout the entire food industry via implementing product improvements to dishes that incorporate this NP by food business operators could be a major key to improving the nutrition of the general population.

In many countries such as France and the Netherlands, governments and food business operators have worked together to improve public nutrition using NPs [12,56,57]. Currently, in Japan, the Ministry of Health, Labor, and Welfare is taking the lead in implementing initiatives to improve the food environment, targeting food business operators [58]. In the future, it will be necessary to use the NPM-DJ (1.0) together with government planners to develop a system that will lead to the improvement of public nutrition in collaboration with industry and government. Currently, some of Japan’s major food business operators are creating their own NPs [44,49]; however, most food business operators do not have NPs; hence, we hope that they will use the NPM-DJ (1.0). If the NP system should be used to encourage product improvement, it is important that nutrient thresholds are kept meaningful and realistic for food developers and nutritionists and that these goals encourage (re)formulation changes and are achievable over time. It is also necessary to evaluate the NPM-DJ (1.0) in actual Japanese settings, such as ready-to-eat meals, home cooking, restaurants, company cafeterias, and school restaurant dishes, and consider how it will be evaluated. To further implement this NPM-DJ (1.0) model to reformulate dishes at restaurants, take-aways, or prepared foods, a guidance for food industries may be needed. The United States Food and Drug Administration issued a guidance document for voluntary sodium reduction [59]. This is based on the analysis of the food supply in 2010 and reflects the current market. A similar approach, such as a comprehensive nutritional analysis of dishes currently consumed in Japan, may be necessary.

In the future, NPs such as the NPM-DJ (1.0), may be recommended and used in public health initiatives in Japan. Moreover, it is necessary to evaluate the long-term impact of the NPM-DJ 1.0, and a prospective cohort study is needed. First, it is necessary to verify the extent to which food manufacturers have used this NP to improve their own products. Next, it is necessary to evaluate the extent to which consumers have accepted products that use the NP and the health effects in the population that has used these improved products.

The NPM-DJ (1.0) has a few limitations. First, when the score distribution within a dish category was narrow, there was no difference in the ratings. Second, the number of target dishes in this study was small; therefore, it was not possible to include dishes that correspond to actual eating habits in Japan. Furthermore, because the final rating results change depending on the number of cooking recipes and changes in the final score, the current rating results do not necessarily reflect the actual eating habits in Japan, and there is a possibility of bias. In the future, we plan to add dishes that represent the dishes and cooking methods across Japan and then classify and evaluate their NPs. Third, because the evaluation was based on one dish unit, the weight of the dish and the type of food selected had a significant impact on the score and rating. In this study, each dish was set up by a dietitian or registered dietitian with experience in creating cooking recipes. To prevent misunderstandings regarding the final scores and rating results for each dish, our recipe information, such as the type of food and weight of each dish, is published in Supplemental Table S2. Fourth, the baseline points, adjustment points, and their balance, as well as the implications of the score ranges of the points and ratings and the scores of the dishes on healthiness, are not clear. In the future, showing the health of the score will reveal the potential impact on the model’s effectiveness.

## 5. Conclusions

In this study, we developed the NPM-DJ (1.0), which assesses each dish to promote health and prevent NCDs in healthy Japanese adults. The use of the Japanese version of the NP proposed in this study by food business operators could potentially trigger improvements and the reformulation of nutrients in dishes (products), leading to a food environment in which consumers can choose healthier foods.

## Figures and Tables

**Table 1:** Dish category classification of the Nutrient Profile Model for Dishes in Japan version 1.0.

Step	Dish Category	Characteristics of the Category	Example Dishes
1	Staple dish	If the dish contains more than 2/3 weight of the following ingredients: rice, bread, noodles, or other grains. One serving (SV): 40 g of carbohydrate derived from the main ingredient.	Rice, bread, udon noodles, zaru soba noodles, ramen.
	Side dish	If the dish contains more than 2/3 (weight) of the following ingredients: vegetables, potatoes, legumes other than soybeans, mushrooms, seaweed, and nuts and seeds. One SV: the weight of the main ingredients is 70 g.	Chilled tomatoes, boiled spinach with soy sauce, vegetable soup, potato salad, simmered hijiki seaweed.
	Main dish	If the dish contains more than 2/3 (weight) of the following ingredients: meat, fish, eggs, soybeans, and soybean products. Six grams of protein from the main ingredient is equivalent to one SV.	Yakitori chicken thigh skewer with tare sauce, sashimi, fried cod, chilled tofu, tamagoyaki (Japanese-style thick omelet).
2	Mixed dish	The main ingredient of the dish does not exceed 2/3 (weight). Staple food with an SV of less than 0.5 + main dish and side dish both with an SV of 0.5 or more.	Nikujyaga (Japanese-style meat and potato stew), sukiyaki, oden, cabbage rolls, sweet and sour pork.
	Mixed dish with staple	The main ingredient of the dish does not exceed 2/3 (weight). The staple food contains an SV of 0.5 or more + at least one of the main dishes and side dishes contains an SV of 0.5 or more.	Fried pork cutlet bowl with egg, Japanese-style curry and rice, hamburger, yakisoba (fried noodles), takoyaki (octopus balls).

**Table 2:** Rating by dish category.

	Dish Category				
Rating *	Staple Dish	Side Dish	Main Dish	Mixed Dish	Mixed Dish with Staple
★★★★★	−2	−7	−4	8	13
★★★★☆	0	−6	−1	13	16
★★★★	1	−5	0	13	17
★★★☆	3	−4	1	14	17
★★★	6	−3	2	16	19
★★☆	9	−1	3	18	19
★★	15	1	6	19	21
★☆	18	2	11	22	26
★	20	4	19	25	29
☆	-	-	-	-	-
*n*	20	34	21	12	18

* A rating of 0.5 to 5 stars is provided depending on the final score for each dish category. ★ means one star, and ☆ means half star.

**Table 3:** The effect of noodle soup consumption on finale scores.

Dish Name	The Amount of Soup Consumed	Food Weight (Excluding Water)	Total Food Weight	Energy	Saturated Fat	Total Sugars	Sodium	Baseline Points	FVNL Points	Protein	Total Dietary Fiber	Final Score
Udon (thick wheat noodles)	Left all soup	332	473	3	0	2	10	15	0	0	5	10
	Half soup consumed	345	564	4	0	2	13	19	0	0	5	14
	All soup consumed	358	658	4	0	3	17	24	0	0	5	19
Tempura udon	Left all soup	375	524	5	1	2	10	18	0	0	5	13
	Half soup consumed	388	615	5	1	2	14	22	0	0	5	17
	All soup consumed	401	709	5	1	3	18	27	0	0	5	22
Zaru soba (cold buckwheat noodles)	Left all soup	317	362	4	0	1	6	11	0	6	6	−1
	Half soup consumed	321	378	4	0	1	7	12	0	6	6	0
	All soup consumed	337	437	4	0	2	11	17	0	0	6	11
Ramen (soy sauce)	Left all soup	249	316	3	0	0	7	10	0	4	8	−2
	Half soup consumed	262	422	4	0	0	16	20	0	0	8	12
	All soup consumed	275	525	4	0	0	24	28	0	0	8	20
Roasted pork ramen	Left all soup	310	377	4	1	0	11	16	0	0	8	8
	Half soup consumed	323	483	4	1	0	19	24	0	0	8	16
	All soup consumed	336	586	5	1	0	27	33	0	0	8	25
Tenshin noodles (crab omelet on ramen)	Left all soup	451	518	6	7	1	19	33	0	0	8	25
	Half soup consumed	464	624	7	7	1	28	43	0	0	8	35
	All soup consumed	477	727	7	7	1	30	45	0	0	8	37

**Table 4:** Scores and ratings for the Nutrient Profile Model for Dishes in Japan version (1.0).

									Score					Rating *
No.	Dish Category	Dish Name	Food Weight (Excluding Water) (g)	Total Food Weight (g)	Energy	Saturated Fat	Total Sugars	Sodium	Baseline Points	V Points	Protein	Total Dietary Fiber	Final Score	By Dish Category
1	Staple dish	Zengayu (gruel)	200	200	1	0	0	0	1	0	0	0	1	★★★★
2		White rice (small, 100 g)	100	100	1	0	0	0	1	0	0	2	−1	★★★★☆
3		White rice (middle, 150 g)	100	100	2	0	0	0	2	0	1	3	−2	★★★★★
4		White rice (large, 200 g)	200	200	3	0	0	0	3	0	1	4	−2	★★★★★
5		Shiomusubi (salted onigiri rice ball)	100	100	1	0	0	1	2	0	0	2	0	★★★★
6		Nigiri sushi (eight pieces of nigiri sushi) with soy sauce for dipping	364	364	6	3	1	9	19	0	0	5	14	★★
7		Tendon (tempura on rice with sauce)	244	256	4	1	0	1	6	0	4	4	−2	★★★★★
8		Chicken rice	297	297	6	8	2	7	23	0	0	6	17	★☆
9		A slice of white bread (six slices in a pack)	60	60	1	1	1	2	5	0	1	3	1	★★★★
10		A slice of toasted white bread (six slices in a pack) with margarine	60	60	2	3	1	3	9	0	1	3	5	★★★
11		A slice of white bread (four slices in a pack)	90	90	2	2	2	4	10	0	2	5	3	★★★☆
12		A slice of toasted white bread (four slices in a pack) with margarine	88	88	3	4	2	4	13	0	0	5	8	★★☆
13		Roll of bread (two pieces)	60	60	2	4	1	2	9	0	2	1	6	★★☆
14		Croissant (two pieces)	80	80	4	13	0	3	20	0	0	2	18	★☆
15		Raisin bread	80	80	2	2	0	3	7	0	2	2	3	★★★☆
16		Udon (thick wheat noodles, all soup consumed)	358	658	4	0	3	17	24	0	0	5	19	★
17		Tempura udon (all soup consumed)	401	709	5	1	3	18	27	0	0	5	22	☆
18		Zaru soba (cold buckwheat noodles, all soup consumed)	337	437	4	0	2	11	17	0	0	6	11	★★
19		Ramen (soy sauce, all soup consumed)	275	525	4	0	0	24	28	0	0	8	20	★
20		Roasted pork ramen (all soup consumed)	336	586	5	1	0	27	33	0	0	8	25	☆
21	Side dish	Chilled tomatoes	100	100	0	0	0	1	1	7	0	1	−7	★★★★★
22		Salted boiled edamame (green soybeans)	49	49	0	0	0	2	2	7	1	3	−9	★★★★★
23		Coleslaw salad	62	62	0	0	0	2	2	5	0	1	−4	★★★☆
24		Lettuce and cucumber salad	92	92	0	0	0	0	0	6	0	1	−7	★★★★★
25		Boiled broccoli salad	118	118	0	1	0	0	1	4	1	5	−9	★★★★★
26		Cucumber with hishio-miso (fermented soybeans, barley or wheat, and vegetable with koji mold)	86	86	0	0	0	1	1	6	0	1	−6	★★★★☆
27		Namasu (shredded vegetables marinated in sweet vinegar)	87	87	0	0	2	7	9	6	0	0	3	★
28		Vinegared dish of cucumber and wakame seaweed	78	80	0	0	0	1	1	7	0	1	−7	★★★★★
29		Boiled spinach with soy sauce	60	69	0	0	0	2	2	6	0	2	−6	★★★★☆
30		Shungiku (garland chrysanthemum) with sesame sauce	72	79	0	0	0	1	1	6	0	4	−9	★★★★★
31		Nasu-shigiyaki (grilled eggplant with sweet miso paste)	153	168	1	1	3	4	9	5	0	4	0	★★
32		Kinpiragobou (stir-fried and braised burdock root and carrot)	80	80	0	0	1	3	4	4	0	5	−5	★★★★
33		Stir-fried cabbage	169	169	1	1	1	4	7	6	0	5	−4	★★★☆
34		Chinese-style stir-fried spinach	121	121	0	1	0	5	6	5	1	3	−3	★★★
35		Stir-fried bean sprouts and chives	84	84	0	1	0	2	3	6	0	2	−5	★★★★
36		Stir-fried and braised komatsuna (Japanese spinach mustard)	119	182	0	0	2	4	6	5	0	4	−3	★★★
37		Kiriboshi-daikon no nimono (simmered dried radish strips)	147	210	1	1	2	4	8	4	1	6	−3	★★★
38		Glazed carrot	66	132	0	1	2	1	4	6	0	2	−4	★★★☆
39		Kabocha no nimono (simmered sweet pumpkin)	111	166	1	0	5	2	8	5	0	5	−2	★★☆
40		Nishime (simmered root vegetables and konjac)	143	220	0	0	1	3	4	1	0	6	−3	★★★
41		Assorted vegetable tempura (with tempura sauce)	140	178	2	2	2	7	13	2	0	5	6	☆
42		Vegetable soup	65	205	0	0	0	4	4	7	0	1	−4	★★★☆
43		Corn soup	143	143	1	2	1	4	8	1	1	0	6	☆
44		Root vegetable soup (miso soup with lot of toppings)	86	286	0	0	1	9	10	4	1	4	1	★★
45		Steamed sweet potato	98	98	1	0	5	1	7	0	0	3	4	★
46		Simmered potato with sweet and salty sauce	148	215	1	0	2	5	8	0	0	4	4	★
47		Simmered satoimo (Japanese taro)	126	197	0	0	1	1	2	0	0	0	2	★☆
48		Potato salad	94	94	1	2	0	1	4	0	0	2	2	★☆
49		French fries	73	73	1	0	0	2	3	0	0	4	−1	★★
50		Miso soup with potato and onion	84	234	0	0	0	4	4	0	0	2	2	★☆
51		Simmered sweet pinto bean	62	62	1	0	10	0	11	0	0	1	10	☆
52		Butter-fried mushrooms	80	80	0	7	0	2	9	5	0	1	3	★
53		Seaweed and tuna salad	134	134	1	2	0	3	6	3	2	0	1	★★
54		Simmered hijiki seaweed	140	188	0	0	1	3	4	5	0	5	−6	★★★★☆
55	Main dish	Yakitori chicken thigh skewer with tare sauce (two skewers)	59	59	1	3	1	2	7	0	4	0	3	★★☆
56		Karaage (Japanese-style fried chicken)	77	77	1	0	0	2	3	0	10	0	−7	★★★★★
57		Pork cutlet (with sauce)	123	123	4	13	1	5	23	0	0	0	23	☆
58		Sauteed Vienna sausages	46	46	2	8	0	3	13	0	0	0	13	★
59		Beef steak	157	157	4	13	0	2	19	0	0	0	19	★
60		Sashimi (sliced raw fish)	61	61	0	1	0	1	2	0	5	0	−3	★★★★☆
61		Seared bonito	88	88	1	1	0	1	3	0	7	0	−4	★★★★★
62		Dried fish (horse mackerel)	40	40	0	2	0	2	4	0	3	0	1	★★★☆
63		Salt-grilled salmon	60	60	1	2	0	4	7	0	5	0	2	★★★
64		Salt-grilled pacific saury	51	51	1	3	0	2	6	0	4	0	2	★★★
65		Yellowtail teriyaki	71	71	2	5	0	2	9	0	6	0	3	★★☆
66		Meuniere salmon	63	63	1	3	0	2	6	0	6	0	0	★★★★
67		Simmered fish (mackerel)	69	82	1	5	1	3	10	0	4	0	6	★★
68		Fried cod	53	53	1	1	0	1	3	0	4	0	−1	★★★★☆
69		Fried egg	52	52	1	2	0	1	4	0	2	0	2	★★★
70		Tamagoyaki (Japanese-style thick omelet)	83	103	1	4	1	3	9	0	3	0	6	★★
71		Scrambled eggs (omelet)	124	124	2	13	0	4	19	0	0	0	19	★
72		Japanese-style steamed egg custard	60	126	0	0	0	4	4	0	3	0	1	★★★☆
73		Chilled tofu	159	159	1	1	0	2	4	0	3	1	0	★★★★
74		Natto	56	56	1	1	0	1	3	0	3	6	−6	★★★★★
75		Mapo tofu	200	227	2	5	1	5	13	0	0	2	11	★☆
76	Mixed dish	Ginger fried pork (with broccoli, tomatoes, and lettuce)	175	175	3	11	0	2	16	0	0	2	14	★★★☆
77		Hamburger steak (with sauce and lettuce)	154	154	3	9	2	7	21	0	0	1	20	★☆
78		Nikujyaga (Japanese-style meat and potato stew)	274	341	3	6	3	5	17	0	0	4	13	★★★★☆
79		Japanese-style cream stew (chicken and vegetable)	338	388	3	8	4	5	20	0	0	2	18	★★
80		Sukiyaki	371	414	7	17	4	10	38	0	0	6	32	☆
81		Sweet and sour pork	272	285	5	12	5	8	30	1	0	4	25	☆
82		Cabbage rolls	366	491	2	5	6	14	27	4	0	0	23	★
83		Assorted tempura (with tempura sauce and grated daikon radish)	239	277	5	5	2	8	20	0	0	5	15	★★★
84		Simmered deep-fried vegetable tofu	134	155	1	2	1	2	6	1	3	3	−1	★★★★★
85		Oden	386	702	2	1	2	13	18	0	0	1	17	★★☆
86		Potato croquette (with sauce)	190	190	4	7	1	5	17	0	0	4	13	★★★★☆
87		Deep-fried horse mackerel, marinated in sweet vinegar sauce	109	117	2	2	3	5	12	0	5	0	7	★★★★★
88	Mixed dish with staple	Oyakodon (seasoned and cooked chicken and egg bowl)	358	407	5	3	4	8	20	0	0	4	16	★★★★☆
89		Fried pork cutlet bowl with egg	442	513	9	13	4	11	37	0	0	5	32	☆
90		Japanese-style curry and rice	453	575	7	6	3	9	25	0	0	7	18	★★★
91		Chinese-style fried rice	350	350	6	6	1	8	21	0	0	4	17	★★★★
92		Shrimp pilaf	329	329	5	10	1	9	25	0	0	6	19	★★☆
93		Bibimbap (Korean rice dish)	404	404	7	9	3	11	30	0	0	4	26	★☆
94		Unaju (eel dipped and broiled in soy-based sauce on rice)	307	307	7	7	2	8	24	0	0	4	20	★★
95		Assorted sandwich (egg, ham, cheese, cucumber, and lettuce)	227	227	5	15	2	9	31	0	0	5	26	★☆
96		Hamburger	159	159	4	8	3	7	22	0	0	3	19	★★☆
97		Hot dog	147	147	3	7	3	7	20	0	0	3	17	★★★★
98		Pizza toast	162	162	4	11	2	8	25	0	0	4	21	★☆
99		Tenshin noodles (crab omelet on ramen, the ramen soup all consumed)	477	727	7	7	1	30	45	0	0	8	37	☆
100		Spaghetti (Napolitan, Japanese-style ketchup-based pasta)	333	333	5	3	3	14	25	0	0	8	17	★★★★
101		Yakisoba (fried noodles)	362	362	5	4	3	9	21	1	0	6	14	★★★★☆
102		Okonomiyaki (fried pancake with cabbage and selected toppings)	222	222	5	9	2	4	20	0	0	3	17	★★★★
103		Takoyaki (octopus balls) (10 pieces)	127	277	3	2	2	5	12	0	5	0	7	★★★★★
104		Macaroni gratin	355	355	5	14	3	10	32	0	0	4	28	★
105		Fried gyoza (with sauce)	183	183	3	6	1	7	17	1	0	4	12	★★★★★

* A rating of 0.5 to 5 stars is provided depending on the final score for each dish category. ★ means one star, and ☆ means half star.

## Data Availability

The original contributions presented in the study are included in the article, further inquiries can be directed to the corresponding author and the first author.

## Supplementary Materials

The following supporting information can be downloaded at: https://www.mdpi.com/article/10.3390/nu16173012/s1, Table S1: Score table of the Nutrient Profile Model for Dishes in Japan version 1.0 [8,33,34,35,39]; Table S2: Dish names, food names, food weights, and nutrients.
