# Supplementary material for: Development of a Nutrient Profile Model for Dishes in Japan Version 1.0: A New Step towards Addressing Public Health Nutrition Challenges
Source: Nutrients. 2024 Sep 6;16(17):3012. doi: 10.3390/nu16173012 (PMC11397037; doi:10.3390/nu16173012)
Supplement: Supplementary file 1 [file nutrients-16-03012-s001.zip › Table S1.pdf]

Table S1. Score table of the Nutrient Profile Model for Dishes in Japan version (1.0)

	Energy*1 (kcal)	Saturated fat*2 (g)	Total Sugar*3 (g)	Sodium*4 (mg)
Points	Per 1 dish	Per 1 dish	per 1 dish	Per 1 dish
0	≦ 83	≦ 0.6	≦ 2.1	≦ 103
1	> 83	> 0.6	> 2.1	> 103
2	> 166	> 1.2	> 4.2	> 206
3	> 249	> 1.8	> 6.3	> 309
4	> 332	> 2.4	> 8.4	> 412
5	> 415	> 3.0	> 10.5	> 515
6	> 498	> 3.6	> 12.6	> 618
7	> 581	> 4.2	> 14.7	> 721
8	> 664	> 4.8	> 16.8	> 824
9	> 747	> 5.4	> 18.9	> 927
10	> 830	> 6.0	> 21.0	> 1030
11	> 913	> 6.8	> 24.6	> 1133
12		> 7.7	> 28.2	> 1236
13		> 8.7	> 32.2	> 1339
14		> 9.8	> 36.6	> 1442
15		> 11.1	> 40.8	> 1545
16		> 12.5	> 45.7	> 1648
17		> 14.2	> 50.7	> 1751
18		> 16.1	> 55.7	> 1854
19		> 18.4	> 61.3	> 1957
20		> 21.0	> 67.1	> 2060
21		> 24.1	> 72.7	> 2163
22		> 27.7	> 79.1	> 2266
23		> 31.9	> 85.6	> 2369
24		> 36.9	> 92.0	> 2472
25		> 42.8	> 99.0	> 2575
26		> 49.5		> 2678
27		> 57.4		> 2781
28		> 66.8		> 2884
29		> 77.4		> 2987
30		> 90.0		> 3090

*1: Score band started at 3.75% of 2200 kcal (nutrient reference value (NRV)s (Japan, 2015)) and extended linearly (2-11 points) [39].

*2: Score band started at 7% of food energy (the Dietary Reference Intakes for Japanese (2020 edition)) and extended linearly (2-10 points) and adjusted (11-30 points, weighted average with the HSR value) [39]. Formula for the weighted average is as follows: the Nutrient Profile Model for Processed Foods in Japan version (1.0) (NPM-PFJ (1.0)) (adjusted) = NPM-PFJ (1.0) (extended linearly) × weight/100 + HSR × (100 - weight)/100 [33, 34, 35].

*3: Score band started at 10% of food energy (recommendation, WHO, 2015) and extended linearly (2-10 points) and adjusted (11-25 points, weighted average with the HSR value). Formula for the weighted average is as follows: NPM-PFJ (1.0) (adjusted) = NPM-PFJ (1.0) (extended linearly) × weight/100 + HSR × (100 - weight)/100.

*4: Score band started at 3.75% of 2756 mg (7 g NaCl, the Health Japan 21 (third term), 2023) and extended linearly (2-30 points) [8].

Table S1. Score table of the Nutrient Profile Model for Dishes in Japan version (1.0) (Continue)

Vegetable (V) Points	non-concentrated fvnl*5 (%)
	Per 1 dish
0	< 40
1	≧40
2	≧ 60
3	≧ 67
4	≧ 75
5	≧ 80
6	≧ 90
7	≧ 95
8	= 100

*5 fvnl: total weight of cooked fruits, vegetables, nuts, legumes, including seaweed, seeds, and mushrooms.

	Protein*6 (g)	Dietary fiber*7 (g)
Points	per 1 dish	per 1 dish
0	≦ 3.0	≦ 0.7
1	> 3.0	> 0.7
2	> 5.8	> 1.4
3	> 8.4	> 2.1
4	> 10.8	> 2.8
5	> 13.0	> 3.5
6	> 15.0	> 4.3
7	> 17.0	> 5.2
8	> 19.0	> 6.1
9	> 21.1	> 7.1
10	> 23.6	> 8.4
11	> 26.6	> 9.8
12	> 30.4	> 11.6
13	> 35.3	> 13.8
14	> 41.6	> 16.6
15	> 50.0	> 20.0

*6: Score band started at 3.75% of 81 g (NRVs (Japan, 2015)) and adjusted (2-15 points, weighted average with the HSR value) [39]. Formula for the weighted average is as follows: NPM-PFJ (1.0) (adjusted) = NPM-PFJ (1.0) (extended linearly) × weight/100 + HSR × (100 - weight)/100 [33].

*[7]: Score band started at 3.75% of 19 g (NRVs (Japan, 2015)) and extended linearly (2-5 points) and adjusted (6-15 points, weighted average with the HSR value). Formula for the weighted average is as follows: NPM-PFJ (1.0) (adjusted) = NPM-PFJ (1.0) (extended linearly) × weight/100 + HSR × (100 - weight)/100 [8, 33, 34, 35].
